# Secret messaging with endogenous chemistry

**DOI:** 10.1038/s41598-021-92987-2

**Published:** 2021-07-06

**Authors:** Eamonn Kennedy, Joseph Geiser, Christopher E. Arcadia, Peter M. Weber, Christopher Rose, Brenda M. Rubenstein, Jacob K. Rosenstein

**Affiliations:** 1 School of Engineering, Brown University, Providence, RI USA; 2 Department of Chemistry, Brown University, Providence, RI USA

**Keywords:** Mass spectrometry, Cheminformatics, Electrical and electronic engineering, Mass spectrometry, Information technology, Nanobiotechnology

## Abstract

Data encoded in molecules offers opportunities for secret messaging and extreme information density. Here, we explore how the same chemical and physical dimensions used to encode molecular information can expose molecular messages to detection and manipulation. To address these vulnerabilities, we write data using an object’s pre-existing surface chemistry in ways that are indistinguishable from the original substrate. While it is simple to embed chemical information onto common objects (covers) using routine steganographic permutation, chemically embedded covers are found to be resistant to detection by sophisticated analytical tools. Using Turbo codes for efficient digital error correction, we demonstrate recovery of secret keys hidden in the pre-existing chemistry of American one dollar bills. These demonstrations highlight ways to improve security in other molecular domains, and show how the chemical fingerprints of common objects can be harnessed for data storage and communication.

## Introduction

Representing digital data in molecular form offers the potential for extreme physical information density and longevity^1–4^, by mapping information into DNA^5^ as well as other families of compounds^6–9^. The small physical size of molecular datasets has motivated applications where information is hidden in the chemistry of objects^10–12^.

Despite recent advances in the theory of molecular data storage^1^, there has been comparatively little work to understand what new vulnerabilities or avenues of attack could arise in molecular data systems^13,14^. Secrecy systems ought to be designed under the assumption that an enemy can apply unlimited resources to intercept a message^15^, so if molecular steganography is used for security, what properties of chemically embedded covers might alert adversaries or eavesdroppers to the presence of a message?

For example, in DNA data storage^16,17^, the presence of PCR primers, terminus tags, and heavily-amplified oligomers are all detection risks which could indicate the presence of digital data. Commercial biochemical kits and assays open the prospect of manipulation of DNA communications by intermediate actors (e.g. by ‘search-and-replace’ genome editing^18^).

In non-genomic chemical datasets^19,20^, the presence of unusual chemical structures could be used to discriminate an embedded covers from other unassuming objects. Even when common molecules are used, they may carry other noticeable features such as correlated concentration profiles, atypical isotope ratios, or bimodal concentration distributions, which could risk exposing the communication to third parties.

**Figure 1:**
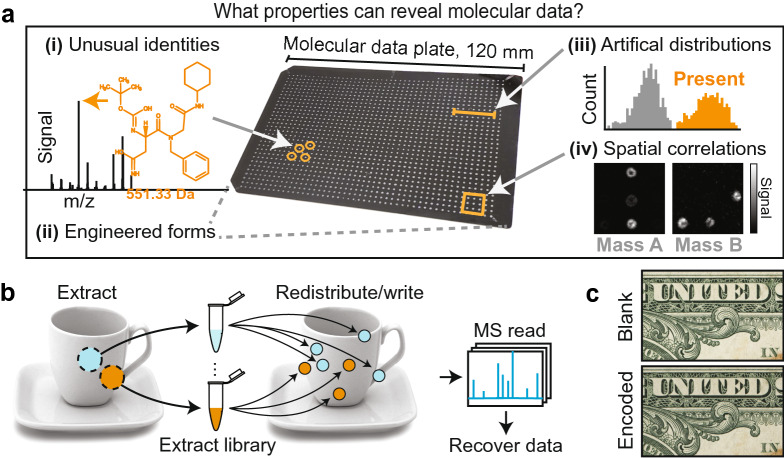
Addressing vulnerabilities of molecular steganography. (**a**) A myriad of non-natural features can expose molecular messages. (**b**) Chemical permutation extracts chemicals from an object, and then redistributes these extracts in non-random spatial patterns to encode data. (**c**) An area of an American one dollar bill, imaged before and after data encoding, showing no apparent modifications.

In this paper, we identify common vulnerabilities of molecular data systems (Fig. 1a), and then experimentally demonstrate a proof of concept chemical permutation framework, which can conceal digital data in pre-existing surface chemistry (Fig. 1b). We elected to encode messages using banknotes as covers (American one dollar bills), but the process does not rely on any specific chemistry, and could be implemented on many common objects.

Our aim was to demonstrate that the existing chemical inhomogeneity in the original substrate can be used to hide information. This is done by permuting pre-existing chemistry in ways that make it analytically indistinguishable from the original substrate (Fig. 1c). Rather than introducing exogenous chemical compounds, we extract endogenous chemical profiles from the object, and then redistribute these samples across the surface in a pseudorandom pattern which encodes digital data. As a result, each bit of information is spread across thousands of pre-existing compounds, embedded into the object’s chemical background variations. The permutation of background noise is a routine steganographic method for embedding hidden data^21,22^, but its conceptualization and demonstration in chemistry provides a means to approach the standards of modern steganography in a molecular system^23–25^. Intriguingly, since the extracts are mixtures whose contents are not known a priori, we can write and recover a hidden message without ever specifying the data-encoding chemistry.

These demonstrations represent a new conceptualization of molecular data encoding which can begin to approach the standards of modern steganography, and improve the robustness of hidden messages against third-party analytical detection, with broad security implications for molecular data and devices.

## Results

### Characterizing the untreated chemical background

**Figure 2:**
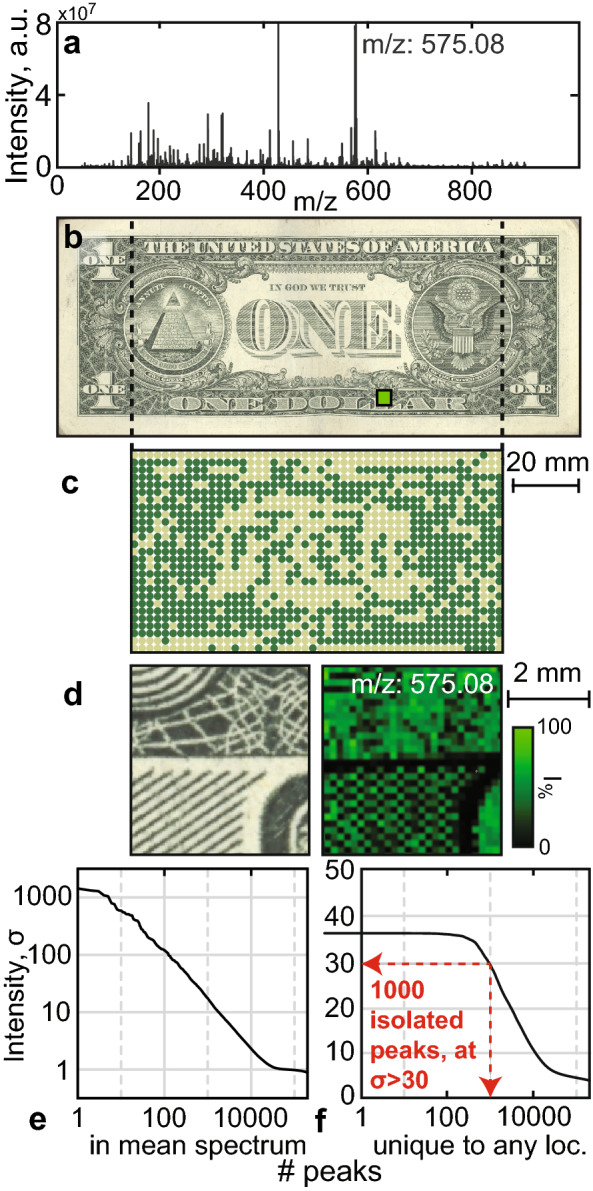
Analysis of an untreated American dollar bill. (**a**) The spectrum average of 1296 locations acquired across the bill’s surface. About 50 prominent dye peaks, and > 1000 trace compounds are evident. (**b**) An optical scan of the dollar bill surface. (**c**) An array of spectral acquisition locations corresponding to the scan image (dotted lines), recorded at 2.3 mm pitch, color coded by cluster. (**d**) Optical image (left) and mass image (right) of a 4.35 mm subsection resolved at 30 $\times$ 30 pixels ((**c**), green square) showing the intensity of a phthalocyanine dye (m/z = 575.08). (**e**) A plot of the peak intensities observed in the mean spectrum, sorted by peak intensity. (**f**) A plot of the number of masses found uniquely in any one location, as specified by the y-axis peak intensity cutoff.

Chemical imaging of an untreated dollar bill (Fig. 2) was performed with a Fourier-transform ion cyclotron resonance mass spectrometer (FT-ICR MS, SolariX 7T, Bruker). The mass spectrum of 1296 surface locations was recorded from 150 to 900 m/z at a sampling pitch of 2.3 mm. The average spectrum of all locations (Fig. 2a) displays a prominent peak at 575.079 m/z, which was identified as a phthalocyanine dye using Tandem Mass Spectrometry (MS/MS). We use this peak as an internal mass shift-reference for each spectrum. We also normalize each spectrum by its standard deviation ($\sigma$), which allows for the comparison of signal strengths across the scanned region (Fig. 2b).

To gain an intuitive understanding of the background spectral diversity, we perform dimensionality reduction of each spectra using Uniform Manifold Approximation^26^ (see Fig. S1). Clustering of the reduced spectra illustrates that the background chemistry broadly falls into two classes, which correlate with the presence/absence of visible dyes across the surface (Fig. 2c), green: dye, tan: absent). A close correlation between visible features and chemical composition remains evident even at sub-mm scales, as illustrated by MALDI imaging (Fig. 2d).

Most of the cover chemical signal is concentrated within a small number of dye peaks, but the bill also displays a logarithmic tail-off of trace products acquired over time and usage (Fig. 2e). Intriguingly, many of these trace elements appear at only one location. About 10,000 peaks (0.13% of the full spectrum) are isolated to a single mm-scale surface location (Fig. 2f). Even allowing for some overcounting of ion adducts, this suggests there are thousands of spatially concentrated trace compounds, which we can observe directly (Fig. S2).

### Characterizing a library of natural extracts

**Figure 3:**
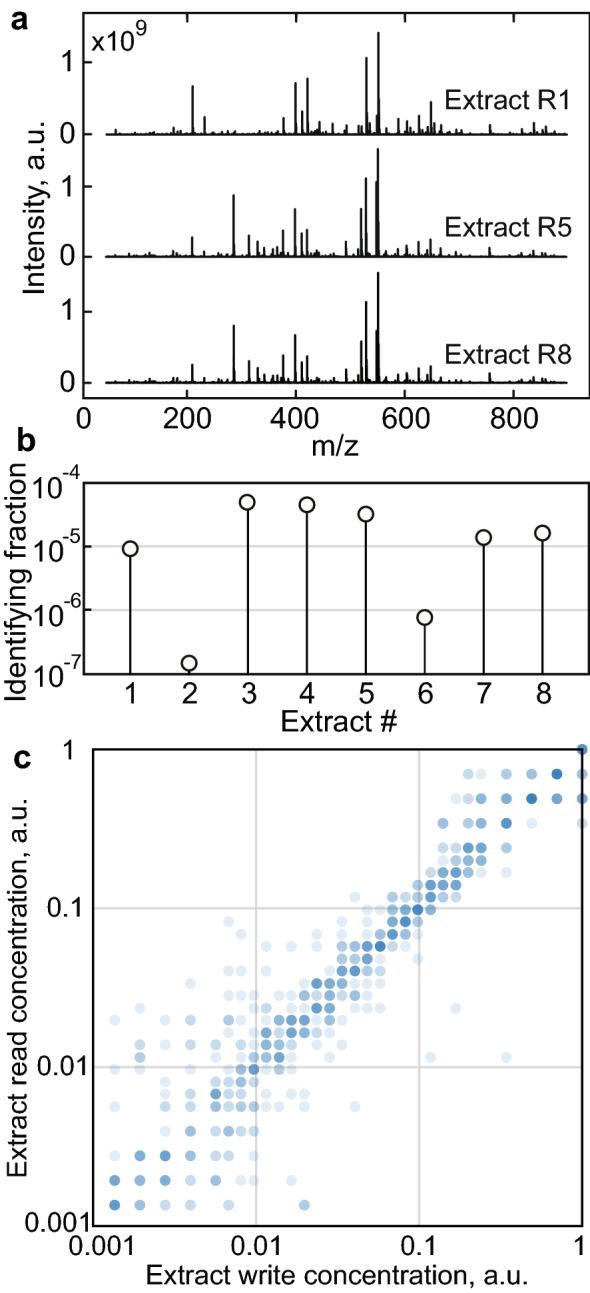
Signal characterization of natural product mixtures and readout. (**a**) FT-ICR spectra of three dollar bill extracts dried onto a steel plate. Each extract is from the same bill. Spectral similarity is qualitatively evident. (**b**) Bar graph quantifying the ratio of identifying compounds to all compounds in each extract, indicating 99.99% of the compounds across extracts are the same. (**c**) Write concentration vs. predicted MS read concentration for 768 extract readings across 32 unique concentration levels, showing the spread of error after multi-mass regression. Observation count increases with color intensity.

Encoding the data begins with the extraction of natural samples from an object. Briefly, to generate each extract, a solvent was manually aspirated from a millimeter-scale region of a banknote surface, and the contents of the solution were isolated and stored (see “Materials and methods” section). Eight samples were extracted from one dollar bill and analyzed by mass spectrometry, three of which are shown for comparison (Fig. 3a). The majority of chemical contents are the same across extracts, and only a small fraction ($\approx$ 0.01%) of the peaks in the mass spectra are unique to one of the eight extracts (Fig. 3b).

### Chemical permutation

To write data, an automated liquid handler (Labcyte, Echo 550) is used to dispense droplets of the extracts back onto the cover surface. In the simplest scheme, the presence (‘1’) or absence (‘0’) of an extract can encode one bit of data per location. Overall, the presence/absence of each extract across many locations encodes the whole message. One banknote may contain several thousand spots, each of which may be a mixture of droplets from multiple extracts. After dispensing the droplets, the solvent is evaporated, leaving the chemical contents of the extracts embedded on the banknote surface.

To further obscure the message, we can also encode data using more than two concentration levels. Although it can make the readout more complex, writing with more concentration levels improves concealment by generating softer concentration gradients, while also increasing the maximum possible information density (see “Materials and methods” section).

For example, given $N = 32$ possible concentrations (Fig. 3c), we can write $\log _{2}32$ = 5 bits per liquid transfer. When encoding data using multiple concentrations, the message is interleaved and divided into symbols of length $\log _{2}N$, where each symbol instructs the liquid handler to dispense a particular concentration of a particular extract to one location.

### Reading redistributed chemistry

After alignment, the extract concentrations at each location are estimated (see “Materials and methods” section) and converted back into binary symbols (e.g. for concentration level 31/32, we would read back the symbol $S = [11110]$). The estimated symbols of all locations and extracts are concatenated, forming one long binary string which is de-interleaved (see “Materials and methods” section) to recover the original message.

Write vs. read concentration is shown in Fig. 3c for $N =$ 32 logarithmically spaced write concentration levels. Concentration regression was performed using a random forest model (see “Materials and methods” section), built using spectral intensities as features, and trained with labelled data (see “Materials and methods” section). Naturally, classification errors become more frequent as the concentration levels become more dense. Eventually, the increasing error rate will outweigh the information density gained by allowing more concentration levels.

### Evaluating the obscurity of embedded data

**Figure 4:**
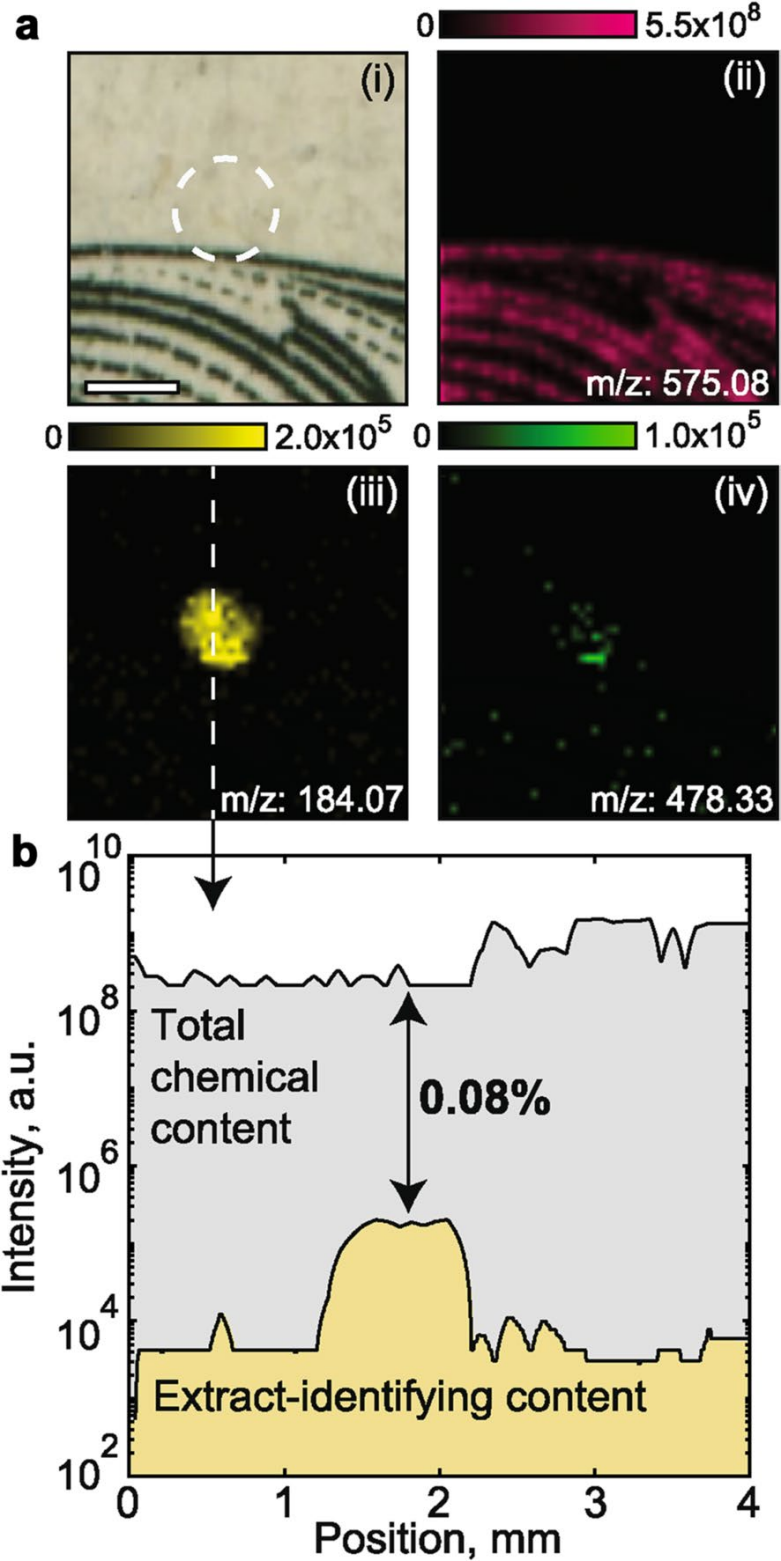
FT-ICR MS imaging of a data-encoded dollar bill. (**a**) The location of a 60 nL deposition of maximum-concentration extract is shown optically (i) and imaged at three masses (ii–iv). (ii) Intensity map at m/z = 575.08, which is a strong background dye (I $\sim 10^{9}$ a.u.). (iii) Intensity map at m/z = 184.07, the most strongly identifying m/z values of the extract and (iv) the second most identifying m/z of the same extract (m/z = 478.33). Scale bar: 1 mm. (**b**) The profile of mass signals across (iii, dotted-white-line), showing the total integrated chemical mass signal (gray), and integrated signal of all masses which can identify the extract (yellow).

An MS imaging survey of an untreated dollar bill (Fig. S3) confirmed that trace elements typically localize at millimeter scales, and account for 0.01–1% of the local chemical diversity. In Fig. 4, we use MS imaging to observe the spatial features of an extract redeposited on a dollar bill. MS imaging shows that the extract deposition spot is clearly defined, but depositions can also track spatially with background chemistry, or may not exceed the background chemical noise. The location of a molecular bit is shown optically (Fig. 4a (i)) and as MS images of several relevant masses (Fig. 4a (ii–iv)). Compared to previous molecular datasets which did not attempt obscurity^9^ (Fig. S4), the data encoding chemistry accounts for only a minute fraction of the location’s total chemical content.

In theory, this particular extract is detectable by MS imaging, but the identifying masses are already present in the context of the object, and their abundance are in line with expectations of a trace product on an untreated bill. Further, the identifying signals are minute against the chemical background of dyes (Fig. 4b, I$_{ext} \sim 10^{5}$ vs. I$_{bkg} \sim 10^{9}$), which would make blind detection of permuted chemistry challenging. A detailed description of ways an attacker could intercept the embedded message are provided in Supplementary Note S1.

### Digital error correction

**Figure 5:**
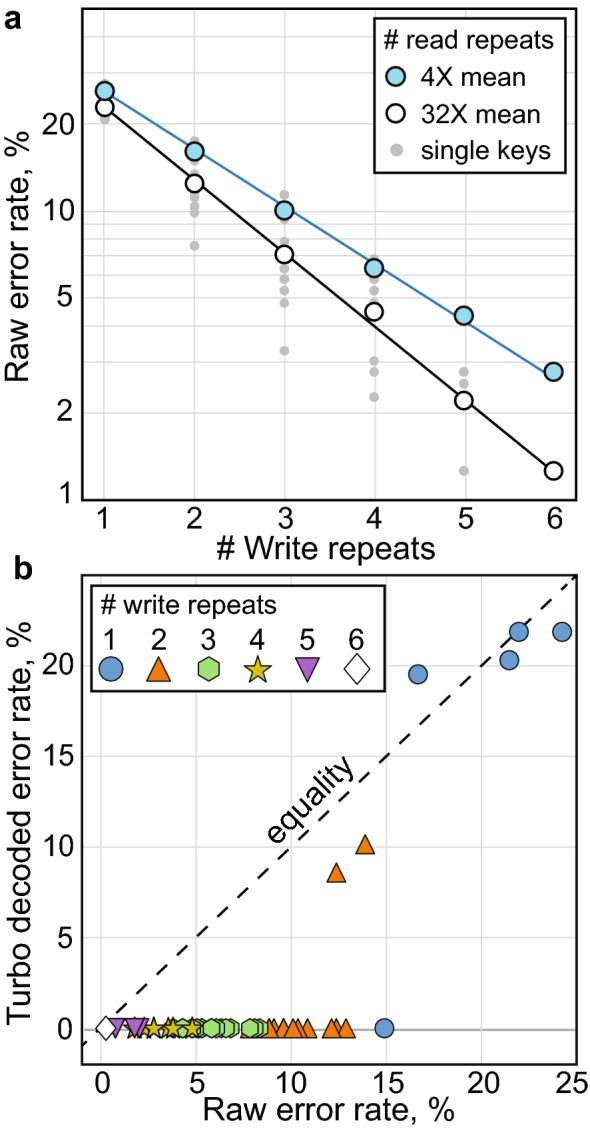
The influence of read/write repetitions on raw/decoded error rates. (**a**) Raw error rates of six messages written to one bill are shown on a logarithmic scale ranging from 1 to 50% as a function of read/write repetitions. Individual keys recovered are shown in gray, and indicate the spread of error. (**b**) Turbo decoded error rates as a function of input raw error rates. Every encryption key is perfectly decoded if the raw error rate is below 12%.

The strategies that hide messages in chemical noise also tend to increase their error rates, which can require molecular error correction^27^. To correct errors, a rate 1/3 Turbo code^28^ was applied to a 128-bit encryption key, producing an encoded payload of 384 raw bits. As an initial demonstration, we used only two concentration levels, and wrote exactly one raw bit per extract, per location. Six repetitions of the coded encryption key (2304 raw bits in total) were interleaved and written onto one banknote. A plot of the raw error rates is shown in (Fig. 5a). The two lines correspond to averaging 4$\times$ (blue) and 32$\times$ (black) mass spectra read from each spot. With no write repetitions, the maximum raw error rate can exceed 20%, which precludes most formal error correcting codes. We are employing the same analysis which achieved just 2% error in a comparable experiment^6^, but the raw error rates are higher here because the extracts are intrinsically difficult to detect against the background. At such high error rates, simple repetition coding is often the optimal outer code^29^ prior to using more sophisticated inner codes which can guarantee perfect message recovery. By integrating the signal from scattered repetitions written across the banknote, the raw error rate is brought well within the tolerance of the turbo code. The key is decoded without error if the raw error rate is 12% or lower after repetition averaging (Fig. 5b).

### Complete workflow

**Figure 6:**
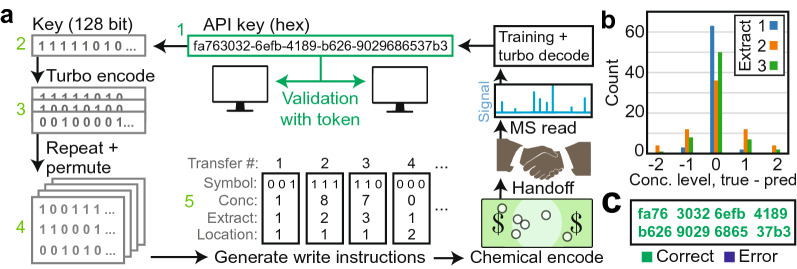
Storage and recovery of an embedded key on a dollar bill. (**a**) Graphical overview of the write and read process. A key is produced which can validate a program (or user) on a new, unauthenticated device (1). The key is binarized (2), turbo encoded (3), padded and interleaved (4), and converted into symbolic liquid transfer instructions (5). The API key can be recovered from the dollar bill using MS analysis and turbo decoding to validate a program or user on an unconnected device. (**b**) Bar graph results of the distance (error) between the four true and predicted concentration levels for the API key written. (**c**) The API key recovered after turbo decoding.

The chemical permutation read and write procedure is formalized and shown graphically in Fig. 6a. We begin with an application programming interface key (API key) which is a shared secret used to authenticate a program (or user), although the choice of data is arbitrary, and any other data of similar size could have been used. The key is preprocessed (Fig. 6a (1–5)) before chemical encoding, which involves binary conversion (2), turbo encoding (3), repetition with interleaving (4), and conversion of the bit string into liquid handler instructions (5). The automated liquid handler dispenses extracts to the banknote, which is handed off, and read using MS for data recovery (Fig. 5b).

Using this procedure, an API key (fa763032-6efb-4189-b626-9029686537b3) was written using three extracts and four concentration levels. Most of the concentrations were correctly identified (Fig. 6b), and after decoding the key was recovered without error (Fig. 6c).

## Discussion

Molecular data can complement traditional data systems with interesting new dimensions and features, including covert messaging. By exploring how patterns in molecular datasets reveal the existence of the data, we offer a new way to think about the connections between analytical limits, natural occurring chemical diversity, and engineered information systems.

In previous work, we encoded data using metabolites^6^ and Ugi reaction products^9^, illustrating how small-molecule chemical diversity can be harnessed for information storage. Although these reports projected molecular data into a broad chemical space, the chemistry was explicitly defined, and each binary value mapped to one compound. Here, we have extended these concepts by encoding information using extracts whose information-bearing chemistries are never explicitly identified. Each bit of information is encoded as subtle shifts in mixture composition, hidden across thousands of naturally-occurring trace compounds.

A perfect secrecy system may be defined as one where the enemy is no better off after intercepting any amount of material than before^15^. Here, since the chemical profile of every object is unique and no exogenous chemistry is introduced^30^, an intercepted message will not yield identifying molecules, although it could yield identifying patterns or correlations. Decoding the data does still require that the sender and receiver share knowledge of reference coordinates (see “Materials and methods” section). Absent this key, the whole banknote must be analyzed, which would take several days, and would still leave a very challenging decoding problem without training labels or spatial registration marks.

These demonstrations offer lessons that can be applied to molecular steganography in other domains. For example, to improve DNA data secrecy, data could be hidden within naturally-ocurring genomes^31,32^, or encoded as genomically plausible sequences. Although minimizing spatial addressing is often considered a feature of DNA data storage, incorporating more spatial encoding in a DNA dataset (multiple spatially separated pools of sequences) could also make it more secure.

By leveraging the chemical diversity and uniqueness of everyday objects, we have shown how molecular data can expand beyond archival data storage, and can offer ways to represent information in low-cost, rewritable, and perhaps even undetectable forms. As molecular information becomes more common, it is valuable to think about ways that messages can not only be protected through obscurity, but can also be made intrinsically resistant to discovery by sophisticated analytical tools.

### Limitations

Coding improves instrumental detection, but it also makes the message more detectable. There are known tradeoffs between detectability and payload size^33^ which would be a valuable direction for future work to explore in chemical space. It is theoretically possible to attack a chemical-permutation encoded object by brute force, although it would be extremely challenging in practice (see Supplementary Note S1). The user also has finite attempts to read the message, since MS ablates material during measurement, and the embedding is progressively degraded. In prior work, we estimated that a dataset could be read 100 times before appreciable degradation^9^, but this study involves trace concentrations in a large chemical background, so the number of read attempts before information loss is likely lower.

At our standard grid pitch, a single dollar bill can hold 2050 data spots, although encoding at higher spatial frequency is achievable. If each spot contains 60 nL, and 2.5 nL is dispensed per extract, we expect a payload limit of about 50,000 raw bits per bill. It is instructive to consider this payload in the context of the pre-existing information content, since chemical media are dense information carriers^34^. The resolving power of our mass spectrometer is on the order of $10^5{-}10^6$. Our imaging experiments (see Supplementary Figs. S3, S4) demonstrated unique spatial features well below 0.1 mm resolution, so at least $10^6$ unique locations are addressable. Therefore, the pre-existing information content of the substrate is estimated to be $10^{12}{-}10^{13}$ raw bits. Viewed another way, our maximum payload is equivalent to introducing 1 bit of permuted chemical content for every 100 billion bits of pre-existing information. This is very low density, which is encouraging for obscurity, but not for applications where larger datasets are needed. Different objects will provide high or lower payload depending on their available area. Our current bit density is not a hard limit, however, and could be overcome by reducing the spatial pitch of the droplets during liquid handling.

The initial chemical diversity of natural objects may also limit the ability to hide data in chemical permutations. Chemically homogeneous objects are likely to have a smaller proportion of extracts which are uniquely identifiable, which could increase error rates, requiring more overhead. The method may be more appropriate for embedding information into objects whose surfaces normally contain diverse mixtures and spatial distributions of chemical compounds.

## Materials and methods

### Materials and reagents

All data encoding chemistry is made up of pre-existing dollar bill natural products. The only specifiable reagants are the solvents used for extraction: Water, methanol, and dimethylsulfoxide (DMSO). For illustrative purposes, we identified a few dyes and trace substances detected on banknotes by MS.

### Extract preparation

To generate an extract, a dollar bill is flattened on top of a non-absorbant tray. DMSO is pipetted on to a bill surface region (0.1–0.2 mL) and vigorously aspirated for 2–3 minutes. DMSO facilitates a longer drying time than methanol or water, which allows for repeated aspiration and improved extraction quality. Aspiration and collection is repeated for the location and all the contents are added to a 1.5 mL eppendorf. The extract contents are left to concentrate by evaporation down to a few $\upmu$L, and transferred to a 384 well plate for robotic liquid handling.

Extracts are diluted at 32 logarithmic concentration steps and stored under sealed refrigeration. A total of 12 extracts each at 32 unique concentrations makes up one 384 well library plate. To test whole-bill extracts, a bill was rolled up, super-saturated in solvent, and left standing up in an eppendorf. After drying, the residual eppendorf sediment was reconstituted.

### Mass spectrometry

Mass spectra are acquired with a Fourier transform ion cyclotron resonance (FT-ICR) mass spectrometer in positive ion mode (Solarix, Bruker). No matrix is added to the banknote, so the extracts and background of the substrate are ionized together using only laser desorption ionization (LDI). Spectra produced by FT-ICR are particularly high resolution, often reaching peak widths below 0.001 Da. All spectra were aligned by a mass lock at m/z = 575.0788. We found good acquisition settings for dollar bill surface analysis by trial and error. The time between spectral acquisitions ranged from 4 to 9 s depending on our instrument settings. The recommended MS settings using a Bruker FT-ICR instrument for analysis of a dollar bill substrate are laser power: 15%, Laser shots: 200, Frequency: 400 Hz, Laser Focus: Large, Beam width: 0.5 mm, and Averaging: > 4×.

### Alignment

When using a dollar bill as a vector for hidden messages, calculating the data encoding positions is very challenging without prior information, because the diffused, dried extracts exist below the natural variation of background chemical/optical signals. To read data, first, each encoded position must be known, so the reader must have some agreed prior information about the bill. In the example in Fig. 5, a 48 $\times$ 32 grid of co-ordinates was aligned using characteristic fiducial marks on the surfaced; the letter ‘w’ in ‘we’ was grid point X23Y09, the bottom of the large ‘N’ in ‘one’ was grid point X25Y17, and the top of the second ‘L’ in ’DOLLAR’ was grid point X32Y24. Triangulation was performed by inputting these three points into the Bruker software to approximate the position of every other grid point. More complex spatial arrangements could avoid using a grid at all, but we implemented this approach for its simplicity, and because it only requires a small amount of prior information. Specifically, 3 sets of co-ordinates (48 bits) are required, along with information about which side the data is written on (1 bit). This compares favorably to the payload of Fig. 5 (2304 bits).

### Data plate preparation

Experimental details of liquid handling and transfer are described in full elsewhere^9^. Briefly, the data to be written is converted to a string of binary values, and reshaped into an $M \times N$ matrix, where *M* is the number of extracts to be used, and *N* is the whole binary string written with only that extract. If required, the input binary string is concatenated with a small vector of zeros before reshaping to allow for the clean construction of the $M \times N$ matrix. In the simplest coding scheme, the presence (‘1’) or absence (‘0’) of the *m*th value in an extract’s string directly defines whether that extract is dispensed (or not) to each location. Defining an extracts string across *n* locations as $N_{1 \ldots n}$, if $N_{3} = 1$, then that extract is deposited to the 3rd location. Similar decisions are made across all extracts and locations. Once all transfers are complete, the dollar bill is left to dry for about 1 h, and was then either read immediately, or left for a few days before MS analysis.

For variable concentration data, *L* concentration states are possible, and we write $\log _{2}L>1$ bits per extract per location. To implement variable-concentration encoding, serial dilutions of each extract are made up in unique library wells, and a csv file ‘picklist’ of dispense instructions is generated for the Echo liquid handler. The picklist defines which dilution of extract from the library is transferred to each surface location. At our standard pitch, a single dollar bill can hold 2050 data spots. Typical depositions are 2.5 nL dispensed per extract per location.

### Data plate analysis

We convert the raw data files from the instrument into custom HDF5 files, for more efficient querying. To normalize signals across measurements, we often convert the raw intensity values of a spectrum to signal-to-noise ratios (SNR) according to the following shift-and-scale relation: $\text {SNR} = \left( \text {I} - \mu \right) /\sigma$, where *I* is an intensity and $\mu$ and $\sigma$ are the mean and standard deviation of the spectrum’s background.

For multi-peak detection, extract presence was found by applying a regression model trained to identify the spectral features correlated with the extract. To reduce computational overhead, masses whose average intensities were close to the noise floor were discarded,from eight million initial samples per spectra down to about 100,000. The Python library Scikit-learn^35^ was used to construct a random forest regression, typically using a 10/90 train/test split.

Repeated reads were performed to gather statistics. However, the time to acquire and process a large set of objects with MS indicates it may not be feasible to apply the same statistical standards from digital systems to molecular data representations.

### Interleaving

Data written directly as liquid handling instructions will introduce identifiable spatial correlations into the chemical profiles. For example, encoding an image with regions of low and high contrast will encode stretches of low [0, 0, 0...] and high [1, 1,…] concentration. A simple way to solve this problem is to randomize the data using interleaving before restructuring it as liquid handling instructions. The data [0, 0, 0, 1, 1, 1] can be interleaved by the indices [4, 3, 6, 1, 2, 5] to mitigate correlations, producing [1, 0, 1, 0, 0 ,1]. The appropriate de-interleaving indices after data recovery is the argument sort of the interleaving indices, which are [4, 5, 2, 1, 6, 3]. The interleaving indices can be regenerated by the reader using a single integer as the seed.

## Supplementary Information

Supplementary Information.

## Data Availability

The software used in this study is based on code available from the Metabolomics Workbench data repository (study ST001173). Software and data are available from the authors on reasonable request.
