# Supplementary material for: Secret messaging with endogenous chemistry
Source: Sci Rep. 2021 Jul 6;11:13960. doi: 10.1038/s41598-021-92987-2 (PMC8260626; doi:10.1038/s41598-021-92987-2)
Supplement: Supplementary file 1 — Supplementary Information. [file 41598_2021_92987_MOESM1_ESM.pdf]

Supplementary Information for: 'Secret Messaging with Endogenous Chemistry'

Eamonn Kennedy[1], Joseph Geiser[2], Christopher E. Arcadia[1], Peter M. Weber[2], Christopher Rose[1], Brenda M. Rubenstein[2], and Jacob K. Rosenstein[1*]

[1]School of Engineering, Brown University, Providence, RI, USA
[2]Department of Chemistry, Brown University, Providence, RI, USA
* Jacob_Rosenstein@brown.edu

Note S1: Adversarial detection of permuted molecular data.

We assume the attacker suspects that a message is embedded in the chemistry of a dollar bill (the cover), and ask what information, chemical features, or correlations, would cause detection. We declare the message insecure if 1. An attacker gains knowledge of the write conditions (data locations, concentrations, spatial frequencies, etc.); 2. The attacker intercepts the shared key; or 3. If the message is detectable by an intermediary who does not have prior knowledge of the write conditions or the shared key. We define detection to be the measurement of embedded cover signals that are statistically distinct from untreated covers at some rate of error, p_e. The detection process is as follows:

- The attacker measures a set of covers suspected to contain modifications, L_{stego}.
- The attacker measures examples of unembedded covers, L_{cover}.
- The attacker tests L_{stego} for chemical uniqueness with respect to L_{cover}.
- The attacker tests L_{stego} for chemico-spatial correlations with respect to L_{cover}.
- An embedded message is detected if an analysis of L_{stego} rejects the hypothesis that it is representative of the L_{cover} set at some rate of error, p_e, according to the relative entropy between the states.

Prior examples of chemical steganography typically involve the addition of externally applied compounds [1-5], and the message is assumed to be hidden based on the obscurity of the chemistry. However, reliance on obscurity may not meet the standards of modern steganography, because an attacker may detect the message by measuring atypical chemistry (e.g. concentrations, masses, or repeating units that are rarely observed in the L_{cover} set). In contrast, in this work, we permute pre-existing chemistry. This is not a new steganographic concept, but its conceptualization and demonstration in molecular space is closer to the established standards of modern steganography, because an attacker can no longer detect the presence of a message simply by identifying atypical chemistry. Further, we write data in chemical noise gradients, which inhibits the attacker in ways that require distinction from digital/electronic steganography:

1. Unlike a digital image, substrate chemistry can not be read repeatedly, since the gold standard, mass spectrometry (MS), ablates and degrades the existing chemistry (see supplementary material of [6]). This means digital representations of chemical noise will always contain instrumental error, and have limited unique reads.
2. Chemical noise gradients are unbounded, and can not be generalized across objects, because chemistry varies from surface-to-surface across an extreme range of concentrations.
3. The time required to read mass spectral images is fundamentally constrained by the finite availability of global mass spectral instrumentation, which is analogous to (and much lower than) global computational power. The time to acquire a high mass, high spatial resolution mass spectral image of the full surface of a macro object (e.g. a dollar bill) is on the order of days to weeks (see supplemental note S2). The time required to sample many objects is a linear multiple of that time.
4. A chemical object can only be read by one MS device in one location at a time. Therefore, unlike a digital file, mass spectral acquisition of an object can not be distributed across systems, or interrogated in parallel by networked devices to reduce read time.

5. Chemical objects are unique. After embedding of a specific chemical object, there exists no unembedded version of the same object for comparison.

6. Every chemical cover (dollar bill) in circulation is measurably unique from all other bills by some property (e.g. uniqueness of a cover can always be assumed in the distribution of some trace element in some specific spatial pattern). Therefore, we have no reason to believe an unembedded cover will be more or less chemically unique from a set of samples than an embedded cover (e.g. periodicities of pen scratches are anomalous, but not data, and we provide examples of these false positives below in supplementary Figure S3).

Taken cumulatively, these points imply that the attacker must overcome several challenges to detect a hidden message. First, they must empirically quantify a heterogenous set of systems that are not generalizable (point 1,2), at significant time/cost (point 3,4) in the absence of benchmarks or stable metrics for what constitutes a positive signal (point 5,6). We proceed assuming these concerns are resolved by the attacker, although given the current state-of-the-art in analytical chemistry instrumentation, we do not claim to know how this could be achieved in practice.

Chemical uniqueness attacks

In a chemical uniqueness attack, the attacker screens all chemical masses, m_0 ... m_n in each labeled group (L_{cover} vs. L_{steg}) in order to determine the probability that the mass profile of L_{steg} is representative of L_{cover}. The attacker constructs a predictive model to discriminate L_{steg} samples from L_{cover} samples, e.g. by learning the discriminating masses. The attacker also models the discrimination of L_{cover} from other L_{cover} examples to estimate the statistical power of the method.

If L_{steg} is predictable within an accepted range of statistical power and accuracy, then the system is insecure. Chemical uniqueness attacks, as described, would correctly detect hidden messages in the above cited prior reports [1-5], since they rely on the presence of atypical masses and chemical motifs. In contrast, in this work, pre-existing redistributed chemistry will yield no chemically unique signatures, and so chemical permutation is robust against detection by chemical uniqueness attacks.

Spatio-chemical attacks

The attacker may also attempt to observe induced spatial correlations to discriminate the labeled states. In this process, the patterns of all chemical masses, $s_0^{\theta,\phi}$ ... $s_n^{\theta,\phi}$ across the surface of a cover are read. By iteration over k covers, a library of unembedded chemical distributions and correlations is acquired $L_{cover} = s_{0...n}^{\theta,\phi}0$ ... $s_{0...n}^{\theta,\phi}k$. According to our definition of detection, the embedded message is detected if an analysis rejects the hypothesis that L_{steg} is a statistically representative member of L_{cover}.

There are several problems with attempting this type of attack. First, the number of naturally occuring spatial patterns is effectively infinite, and it is unclear what value of k would be needed to representatively sample the full population of all spatio-chemistry. For example, even sampling 10^{-6} of the US dollar bill population would require imaging about 10,000 bills at sub-mm resolution, which would easily use the full supply of globally available mass spectral instrumentation. Still, it is clear that brute force attacks are technically possible. There are other practical concerns that, while trivial, provide clear examples of how the spatio chemical detection could immediately fail. For example, if the spatial frequency of the attackers chemical sampling is less than double the spatial frequency at which the message was written (which is unknown to the attacker), then spatial correlations and periodicities may not be identified. The attacker should therefore choose the smallest instrumentally-possible spatial frequency, although this would further multiply time costs.

Spatial subsampling attacks

The attacker may resort to random subsampling of covers to improve the diversity of detection statistics and reduce time cost. Here, we describe how this can be anticipated and rebuffed by appropriate selection of embedding write parameters. We assume n_{loc} write locations are available on a cover. We assume the attacker has no knowledge of the subset C of co-ordinates of n_{loc} where data is written. Therefore, the probability, p_{sample}, of a randomly sampled location containing data is the ratio of written to unwritten surface areas, i.e. $p_{sample} = C/n_{loc}$ (p_{sample} ∼0.01-0.001 in our experiments). Clearly, the attacker needs enough total encoded samples n_{meas} to observe atypical noise in the distribution of chemical gradients. The minimum required n_{meas} will scale superlinearly with the ratio, r, of written-to-pre-existing concentrations (in our experiments, $r \sim 0.01$). Additionally, subsampling will fail to detect the message if the sender anticipates this form of attack, and writes with sufficiently sparse embeddings. A mathematical description of sparse encoding in molecular data is provided by the authors in [7].

Attack given knowledge of the shared key or write parameters

We estimate the reduction in effort if the attacker has knowledge of the shared key. Given the key, the attacker knows a specific subset of co-ordinates to measure on a putative cover. This reduces the instrumental acquisition time by at least a factor of C/n_{loc} (∼0.01-0.001 in our experiments) per cover tested. The key also provides knowledge of the ordering of spatial locations,

which means just one of $> 10^{300}$ possible orderings needs to be considered during analysis for our reported write conditions. Additionally, the key essentially provides training labels for identifying the information-encoding masses. Therefore, knowledge of the key can be used to disqualify all compounds not detected within the key co-ordinate subset. If all trace elements are randomly distributed, the expectation would be that the attacker can focus on a reduced set C/n_{loc} of all masses.

Note S2: Time cost of brute force attacks.

Data hidden by chemical permutation could be revealed by brute force methods. One method to blindly recover data would be to acquire high-resolution mass spectral (MS) images of the object surface, and search the MS images for unusual sub-background patterns or correlations. If an object is imaged at lower resolution than we reported in the main text (e.g. at 100 samples per mm^2), imaging a whole banknote ($\sim$10,300 mm^2) would require $>10^6$ high-resolution spectra. On our instrument, $>10^6$ spectra would require about $t_{cover} = 30$ days of continual MS instrument operation to complete. On a fast scanning MS system, this would still take several days, and would return higher error rates. The total experimental duration of the attack requiring measurement of k unembedded covers is $t_{net} = k \times t_{cover}/m$, where m is the number of mass spectrometers operating in parallel, e.g. if k covers are required to imaged by $m = k$ systems in parallel, then $t_{net} = 30$ days.

We frequently found fingerprints, nonspecific patterns, textured residues, pen marks, and ink stamps on dollar bills (Figure S3). These are all non-native, patterned chemistries, which could incorrectly be detected as an encoding. It is unclear under what conditions a learning algorithm could discern between actual data-encoding chemical patterns and the normal, patterned chemical residues that objects accumulate over time, handling, and usage.

Supporting figures.

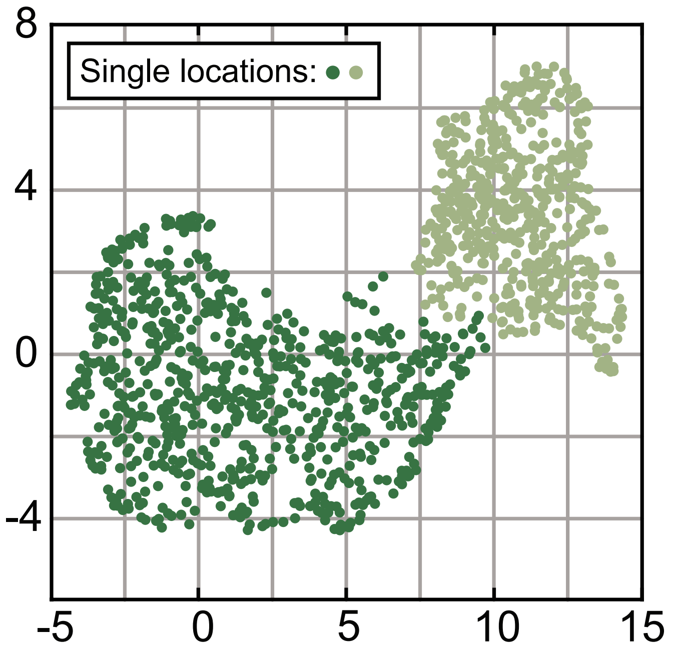

Figure S1. Spatio-chemical analysis of a dollar bill surface using dimensionality reduction. Spectra were recorded for 1296 locations across a dollar bill's surface. Each location's spectrum was reduced down from $>10^6$ masses to a single, bivariate grid co-ordinate using Uniform Manifold Approximation [8]. Broadly, the spectra fall into two clusters which correspond to locations where the primary inks were present (green), or absent (tan).

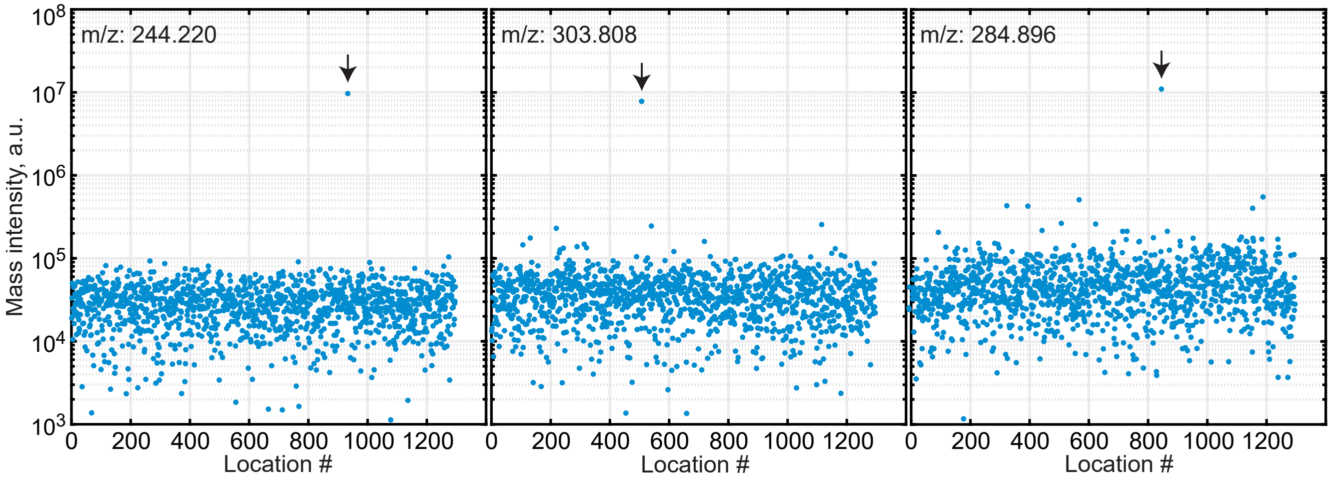

Figure S2. Identifying isolated natural products on banknotes. Circulated banknotes harbor diverse, spatially isolated trace products which are acquired over time and usage. These three plots show the mass spectral intensity at every sampled location of an untreated banknote (1296 total) for three example masses (recorded in the top left corners). In these selected examples, the single masses are intense at only one sub-mm surface region. Thousands of similarly isolated products exist on a single one dollar bill. Approximately 15% of all sampled locations contained at least one entirely unique compound, which is high, and implies significant amounts of spatially-isolated trace products are present on everyday objects.

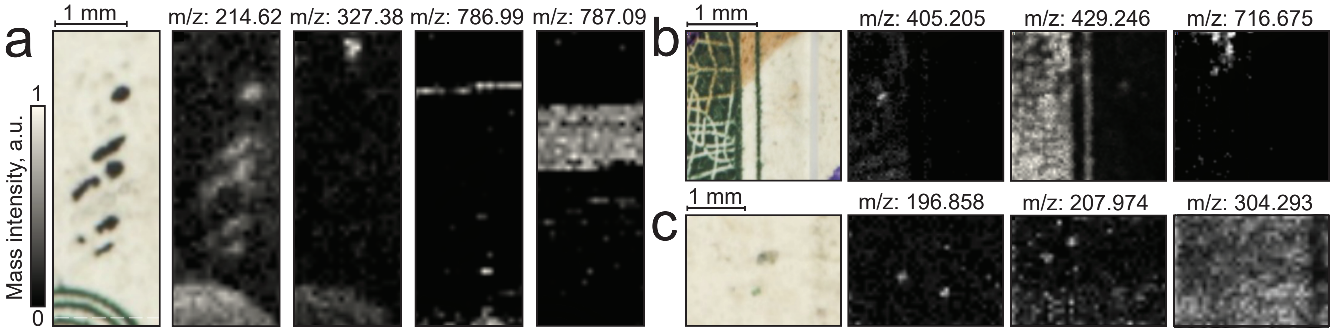

Figure S3. MS-FTICR imaging survey of an untreated dollar bill. A dollar bill surface was imaged optically, and at all masses in positive mode for three mm-scale regions (**A,B,C**). The first mass image shown in (**A**) at 214.62 m/z strongly correlates with the optical image, but the other masses are present in non-optical patterns, and typically localize to 0.1-1 mm-scale regions of the surface. Similar observations of localized trace products were made elsewhere (**B,C**). These features can be mimicked during the write process to conceal molecular data.

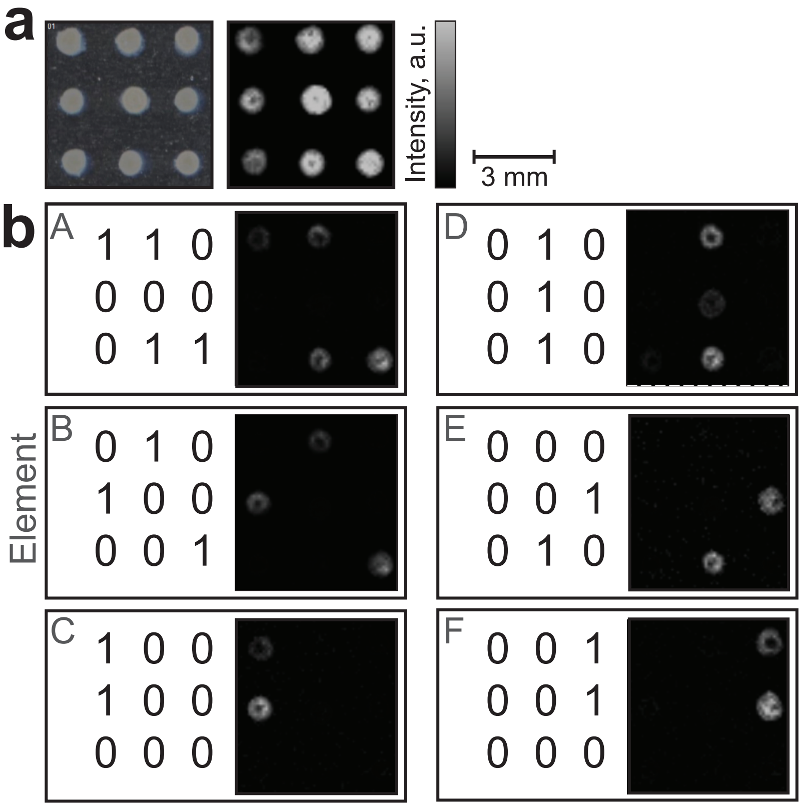

Figure S4. MS-FTICR imaging survey of a molecular data system employing Ugis. **(A)** Optical (left) and MS (right) images of a 3x3 grid of chemical data spots which in total encode 288 raw bits with 32 Ugi products (2). The data is immediately exposed by both optical and chemical detection. MS imaging at six different mass intensities (**B**) can directly recover the message raw bits by revealing the presence/absence patterning of each element. The message is not only detectable, but readable. The message could also be altered by an intermediary actor by adding compounds of appropriate mass to some locations.

References

(1) Andréasson, J. Pischel, U. Molecules for security measures: from keypad locks to advanced communication protocols.Chem. Soc. Rev.47, 2266–2279 (2018).
(2) Sarkar, T., Selvakumar, K., Motiei, L. Margulies, D. Message in a molecule.Nat. communications7, 11374 (2016).
(3) Zhu, Q. Y.et al. Graphene-based steganographically aptasensing system for information computing, encryption and hiding, fluorescence sensing and in vivo imaging of fish pathogens. ACS applied materials interfaces11, 8904–8914 (2019).
(4) Dahn, D. 'molecular' cybersecruity vs. information security. DarkReading (2017).
(5) Boukis, A. C. and Reiter, K. and Frolich, M. and Hofheinz, D. and Meier, M. A. R. Multicomponent reactions provide key molecules for secret communication. Nat. communications 9, 1439 (2018)
(6) Arcadia, C., Kennedy, E., Geiser, J., Dombronski, A., et al. Multicomponent molecular memory. *Nature Communications* 11,616 (2020).
(7) 'Principles of Information Storage in Small-Molecule Mixtures' J.K. Rosenstein et al. IEEE Transactions on NanoBioscience, vol. 19, 378-384, (2020).
(8) McInnes, L., Healy, J. & Melville, J. UMAP: Uniform Manifold Approximation and Projection for Dimension Reduction. arXiv:1802.03426 [stat.ML](2018).
